# Baseline brain and behavioral factors distinguish adolescent substance initiators and non-initiators at follow-up

**DOI:** 10.3389/fpsyt.2022.1025259

**Published:** 2022-12-08

**Authors:** Goldie A. McQuaid, Valerie L. Darcey, Amanda E. Patterson, Emma Jane Rose, Ashley S. VanMeter, Diana H. Fishbein

**Affiliations:** ^1^Department of Psychology, George Mason University, Fairfax, VA, United States; ^2^Center for Functional and Molecular Imaging, Georgetown University Medical Center, Washington, DC, United States; ^3^The Interdisciplinary Program in Neuroscience, Georgetown University, Washington, DC, United States; ^4^Department of Psychology, The Pennsylvania State University, University Park, PA, United States; ^5^Frank Porter Graham Child Development Institute, The University of North Carolina, Chapel Hill, Chapel Hill, NC, United States; ^6^Department of Human Development and Family Studies, The Pennsylvania State University, University Park, PA, United States

**Keywords:** adolescence, anterior cingulate (ACC), decision making, insula, reward, risk-taking, substance use

## Abstract

**Background:**

Earlier substance use (SU) initiation is associated with greater risk for the development of SU disorders (SUDs), while delays in SU initiation are associated with a diminished risk for SUDs. Thus, identifying brain and behavioral factors that are markers of enhanced risk for earlier SU has major public health import. Heightened reward-sensitivity and risk-taking are two factors that confer risk for earlier SU.

**Materials and methods:**

We characterized neural and behavioral factors associated with reward-sensitivity and risk-taking in substance-naïve adolescents (*N* = 70; 11.1–14.0 years), examining whether these factors differed as a function of subsequent SU initiation at 18- and 36-months follow-up. Adolescents completed a reward-related decision-making task while undergoing functional MRI. Measures of reward sensitivity (Behavioral Inhibition System-Behavioral Approach System; BIS-BAS), impulsive decision-making (delay discounting task), and SUD risk [Drug Use Screening Inventory, Revised (DUSI-R)] were collected. These metrics were compared for youth who did [Substance Initiators (SI); *n* = 27] and did not [Substance Non-initiators (SN); *n* = 43] initiate SU at follow-up.

**Results:**

While SI and SN youth showed similar task-based risk-taking behavior, SI youth showed more variable patterns of activation in left insular cortex during high-risk selections, and left anterior cingulate cortex in response to rewarded outcomes. Groups displayed similar discounting behavior. SI participants scored higher on the DUSI-R and the BAS sub-scale.

**Conclusion:**

Activation patterns in the insula and anterior cingulate cortex may serve as a biomarker for earlier SU initiation. Importantly, these brain regions are implicated in the development and experience of SUDs, suggesting differences in these regions prior to substance exposure.

## Introduction

Adolescence is commonly characterized as a period of increased risk-taking coupled with heightened reward sensitivity (1,2). Risk-taking during this evolutionarily conserved developmental period may have positive outcomes (3), with exploratory behaviors allowing for adaptive risk-taking (4–7), facilitating the achievement of key developmental milestones in preparation for the transition to adulthood (8). However, brain changes that condition adaptive risk-taking also render adolescents vulnerable to risk-taking that can lead to negative outcomes, including substance use (SU) (9).

Early SU initiation is associated with a constellation of other negative risk-taking behaviors and related adverse outcomes (10), including delinquency or criminal activity (11), risky sexual behavior (12, 13), physical assault (14), accidental injury (15), and death (16). Given the potential for deleterious outcomes, SU among adolescents has been identified as a global health concern (17). Critically, while earlier SU initiation is associated with greater risk for development of lifetime SU disorders (SUDs) (18–27), any delay in SU initiation decreases risk for development of SUDs (19, 28). For instance, each year of delayed alcohol initiation is associated with a 5–9% decrease in risk for alcohol use disorder (20). Identifying factors which help us to understand - and ultimately predict - early initiation, may be beneficial in targeting prevention efforts to delay SU onset.

Developmental neuroscience models offer potential explanations for increased risk-taking during adolescence that leads to SU initiation. The dual systems (29, 30), triadic (31, 32), and imbalance models (33, 34), generally postulate that subcortical brain regions (e.g., ventral striatum, amygdalae) develop earlier than neocortical regions [e.g., prefrontal cortex (PFC)], and that these asynchronous maturational trajectories condition increased risk-taking in adolescence. Specifically, earlier development of brain structures associated with reward processing, relative to development of neocortical regions associated with cognitive control, generates an imbalance, whereby earlier-maturing reward-processing systems exert greater influence over behavior in adolescence. Development of the PFC and its functional networks, which continues throughout adolescence and into early adulthood (35, 36), is associated with improvements in top-down control of behavior (37–39). This protracted course of development may render the PFC vulnerable to the impacts of abused substances during adolescence (40), with early SU potentially altering neurodevelopmental trajectories (41–44) and, ultimately, adversely affecting adult neurobiology and behavior (9, 45).

Deemphasizing the role of a subcortical-cortical “imbalance” in increased adolescent risk-taking is the Lifespan Wisdom Model (46). This model incorporates fuzzy-trace theory’s conceptualization of risk-related decision making (47) and emphasizes the adaptive nature of increased risk-taking during adolescence. The Lifespan Wisdom Model posits that youth with pre-existing compromised cognitive control form a subset of adolescents who are vulnerable to those risk-taking behaviors with negative sequelae (e.g., addiction).

Given the proximal and distal negative outcomes associated with early SU, it is critical to understand behavioral and neural risk markers that *precede* initiation. Adolescents who initiate SU early often demonstrate pre-existing heightened impulsivity (48, 49), sensation seeking (50), and reward sensitivity (51–53). Such traits are associated with poor emotion regulation (54), behavioral dyscontrol (53), and a relative imperviousness to punishment (55), along with increased susceptibility to negative peer influences (56).

It is key to understand not only these types of behavioral markers associated with increased risk for SU initiation, but also to understand the underlying neurobiology associated with this risk (57). Identifying neurobiological profiles of SU initiation will help identify neuroendophenotypes associated with risk for and protection from SUDs (58, 59). And indeed, brain metrics may predict risk for psychopathology with greater specificity and sensitivity than behavioral measures alone (60).

Existing functional neuroimaging research that has examined neural predictors of SU initiation and/or SU escalation in adolescents reports differences in brain activity in regions implicated in reward processing, including striatum (49, 61–63), amygdala (49), and medial orbitofrontal cortex (64). These studies have not all examined SU broadly, however, with some characterizing initiation/escalation of alcohol only, without examining other substances (49, 63). Additionally, few studies have prospectively investigated brain markers of SU initiation in early adolescent samples comprised only of youth who have not initiated SU. Characterizing neurobiology *prior to substance initiation* is particularly critical, given that substance exposure may alter neurobiology, and that such alterations challenge our ability to disentangle whether brain differences—for example, between those who have and have not initiated SU (or between those with and without SUDs)—are an antecedent or a consequence of SU.

Thus, the central aim of the current study was to comprehensively characterize demographic, behavioral, cognitive, and neural factors that may be associated with risk for substance initiation, including both alcohol and drugs, in a drug- and alcohol-naïve sample. We examined 70 (aged 11.1–14.0 years) SU-naïve early adolescents prospectively over 36 months. We compared those who did and did not report initiation of alcohol and/or drugs at follow-up on “baseline” demographic, behavioral, cognitive, and neural measures. We characterized behavioral and neural profiles associated with reward-sensitivity/risk-taking in relation to SU initiation. Adolescent participants completed measures probing reward sensitivity and risk aversion [Behavioral Inhibition System/Behavioral Activation System (BIS/BAS) Scales], and tasks to assess risk-taking and impulsivity in the context of rewards (Wheel of Fortune and delay discounting tasks, respectively). We predicted that at baseline, those who would go on to initiate SU at 18- or 36-months follow-up would demonstrate greater hedonic and behavioral responsivity to rewards, overvalue immediate rewards, and make riskier choices in a reward-related decision-making task compared to adolescents who remained SU-naïve throughout the study. Further, we predicted that, prior to SU onset, reward-based decision making would be associated with differences between subsequent SU initiators and non-initiators in brain regions implicated in decision-making under uncertainty [i.e., medial prefrontal cortex (65–68)], and in the modulation of reward processing/sensitivity [i.e., ventral striatum and amygdalae (65, 69)].

## Materials and methods

### Study design

Participants were recruited as part of the Adolescent Development Study (ADS), a prospective longitudinal investigation of the neurodevelopmental precursors to and consequences of early SU initiation and escalation. Detailed information on ADS study methods and aims is presented elsewhere (70). Briefly, a total of 135 typically developing, SU-naïve early adolescents were recruited from the Metropolitan Washington D.C. region and followed longitudinally. Demographic, cognitive, behavioral, and imaging assessments were conducted at an initial (“baseline”) visit and during two follow-up visits, at 18.4 (SD = 3.6) months (Wave 2) and 36.7 (SD = 4.4) months (Wave 3) after baseline. Imaging and behavioral data reported here were collected during the initial SU-naïve baseline assessment. Exclusionary criteria for the study included adolescent self-report of alcohol (>1 full drink of alcohol at any time) or, with the exception of nicotine, any SU prior to the initial visit; *in utero* exposure to alcohol or illicit drugs (parent-reported); a diagnosed neurodevelopmental disorder (e.g., autism spectrum disorder); left-handedness; a sibling of a current participant; history of head injury resulting in loss of consciousness >5 min; or MRI contraindication. The Georgetown University IRB approved all procedures, and written consent and assent were obtained from the parent and adolescent, respectively.

#### Participants

Of the 135 participants enrolled in the study, 70 adolescents aged 11.1–14.0 years [*M* = 12.7 years, SD = 0.66; female = 40 (57%)] were included in the analyses reported here. One enrolled participant was excluded due to neurodevelopmental disorder. Participants were excluded from analyses due to missing or incomplete imaging data (*n* = 15) and/or excessive head motion during imaging (*n* = 24). Additionally, since a primary aim was to examine neural activation during risk-taking, participants who did not make any “high-reward/high-risk” selections during the Wheel of Fortune task (WOF; described below) were excluded from analyses (*n* = 4). Groups were defined based on SU status at follow-up, as detailed below. Participants for whom SU status could not be determined due to attrition or survey discrepancies (*n* = 21) were also excluded from analyses reported here. (Supplementary Table 1 provides a detailed summary of exclusions/inclusions).

##### Family/Caregiver measures

###### Socioeconomic status index

An Socioeconomic status (SES) Index was calculated by averaging the mean of two standard scores (mean household income bracket before taxes and mean cumulative years of parental education), and re-standardizing these to obtain a z-score distribution with a 0-centered mean and a standard deviation of 1 for the sample analyzed (*N* = 70) [method adapted from Manuck et al. (71)].

###### Family history of substance use

History of alcohol and SU problems in biological relatives of participants was determined using a modified Family Tree Questionnaire (FTQ) (72), which was completed by the accompanying parent. The FTQ was modified to include drugs of abuse, reported separately from alcohol. Respondents were asked to report alcohol/drug use history of first- and second-degree biological relatives of the enrolled adolescent as follows: 1 = never drank/never used, 2 = social drinker/occasional user, 3 = possible problem drinker/possible problem user, 4 = definite problem drinker/definite problem user. Respondents could also indicate that they did not know or did not remember. Each parent completed an FTQ reporting on the FH for his/her own biological relatives and provided information for the non-visiting parent’s family, where possible. In the analyses reported here, determination of positive FH (FH+) was defined as *possible* or *definite problematic* alcohol or drug use by either the mother or father of the adolescent; otherwise, the adolescent was considered FH negative (FH−).

##### Adolescent measures

###### SU initiation status

At baseline and at the Wave 2 and Wave 3 follow-up visits, adolescents completed two self-report surveys to determine SU status: the Tobacco Alcohol and Drug (TAD) survey and the Drug Use Screening Inventory Revised (DUSI-R) (73, 74). The study-specific TAD included the alcohol and drug portion of the Semi-Structured Interview for the Genetics of Alcoholism (75) and asked about the use of substances, including tobacco, alcohol, and illicit drugs (i.e., marijuana, cocaine, methamphetamine, ecstasy, opiates, salvia, synthetic marijuana, inhalants, and illegally used prescription drugs), along with an open-ended “any other substances” question.

Adolescents also completed the DUSI-R, a survey with demonstrated psychometric validity (76–78) and reliability (79) for assessing SU and factors associated with risk for SUD later in adolescence. The DUSI-R includes 20 questions concerning use of specific substances (e.g., alcohol, marijuana, prescription painkillers, smoking tobacco, chewing tobacco) or substance classes (e.g., over the counter medications, tranquilizer pills, stimulants).

For the purposes of the analyses reported here, affirmative SU responses on both the TAD *and* the DUSI-R were used in determining SU status. Participants who reported SU on both the TAD and DUSI-R at either Wave 2 or Wave 3 follow-up were categorized as SU initiators (SI). Those who reported no SU on both the TAD and DUSI-R at both follow-up assessments were categorized as SU non-initiators (SN). As detailed above, participants for whom SU status could not be determined were excluded from analyses reported here.

As noted above, nicotine use reported at baseline was not considered exclusionary for the current study. Of importance, however, only two participants reported nicotine use, one in each of the SU groups (both reporting last use >30 days prior to the baseline visit).

###### DUSI-R absolute problem density (APD) score

In addition to questions concerning SU, the DUSI-R probes experiences and behaviors known to precede and co-occur with SU. The survey includes eight domains comprised of 159 *yes-no* items that are relevant for early adolescents: SU, behavior, health, social competence, psychiatric symptoms, school performance, family and peer relationships, and recreation (80). An absolute problem density (APD) score, which reflects overall risk for SU, is calculated by dividing the total number of “yes” questions by the total number of DUSI-R items. Here, group comparisons were conducted for the DUSI-R APD score only.

###### Delay discounting (DD) task

Adolescents completed the delay discounting (DD) (81) task outside of the scanner. The task was implemented in E-Prime 2.0. Participants were instructed to choose between receipt of a variable immediate reward (≤$10, in increments of $0.50), versus receipt of a fixed $10 after a specified temporal delay (e.g., *Would you rather have $2 now, or $10 in 30 days*). Discounting was assessed at six delays: 1, 2, 10, 30, 180, and 365 days. Participants were instructed to make their selections with care, as they would receive a reward (≤$10) based on a random selection of one of their choices (82).

Values for which the participant demonstrated equal preference for immediate versus delayed receipt (i.e., the “indifference point”) were normalized to the fixed delayed reward value ($10) (83) and plotted against each delay. To adjust for unequal weighting of indifference points at longer delays (a limitation of conventional methods of calculating area under the discounting curve; AUC), while preserving the notion of subjective experience of time *via* delay scaling (an appeal of conventional AUC metrics), data were log10-transformed [AUClogd (84)]. Values ranged from 0 to 1, with smaller AUClogd values representing steeper discounting and thus preference for immediate (smaller, sooner) reward.

###### Behavioral Inhibitory System/Behavioral Activation System (BIS/BAS) Scale

Adolescent participants completed the BIS/BAS (85), a 20-item self-report measure answered on a 4-point Likert scale. The 7-question BIS scale probes behavioral and emotional responsivity to punishment. Conversely, the BAS is comprised of three sub-scales: Reward Responsiveness, Drive, and Fun Seeking. A higher BIS score reflects aversion to and avoidance of potential punishment, while higher BAS sub-scale scores reflect positive emotionality (Reward Responsiveness) and behavioral approach (Drive and Fun Seeking) in the context of potential rewards.

###### IQ and pubertal development measures

Full-scale IQ (FSIQ) was estimated using the Kaufman Brief Intelligence Test (KBIT), Second Edition (86). Adolescents completed the Pubertal Development Scale (PDS) (87, 88) as a proxy assessment of physical development *via* Tanner stage (87).

###### Wheel of Fortune (WOF) task

**FIGURE 1:**
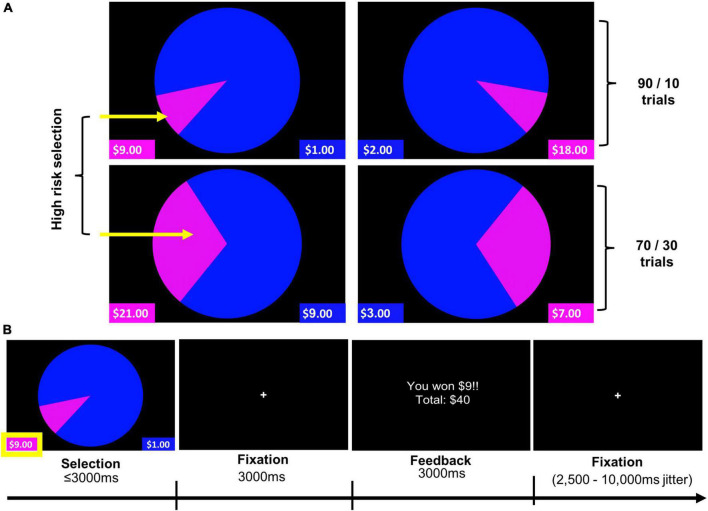
Wheel of Fortune (WOF) task. **(A)** Task stimuli illustrating two trial types (90/10 and 70/30), and highlighting high-reward/risk selections for each of these trial types. **(B)** Example trial timing. Pressing the left button, corresponding to the smaller, magenta portion of the wheel, represents selection of the high-reward/risk option (10% chance of receiving $9), over the low-reward/risk option depicted in blue (90% chance of receiving $1). Analyses reported examined Selection and Feedback phases of the task.

The WOF task was completed during functional neuroimaging. This well-validated paradigm has been used to probe the neural bases of reward responsivity and risky-decision making under conditions of probabilistic reward versus penalty in both adults (66, 67, 89, 90) and adolescents (66, 90, 91). A modified version of this task was used in this study to probe reinforcing outcomes (i.e., winning or losing) (Figure 1; see Supplementary section 1.1 for further description of the WOF task).

Participants were guided through an in-scanner practice (during the structural MRI scan) to ensure their understanding of how to perform the task. Prior to each run, participants were encouraged to maximize their hypothetical gains and/or exceed their previous total winnings. The task was implemented in E-Prime 2.0, and stimuli were presented *via* back-projection onto a screen viewed in a mirror mounted to the head coil. A slow event-related design with temporal jitter provided by a variable inter-trial fixation of 2,500–10,000 ms based on a Poisson distribution was utilized.

Contrasts of interest for the selection and feedback phases were High-reward/risk > Low-reward/risk and Win > Lose, respectively. Behavioral data analyses considered the percentage of high-reward/risk selections and the average response times (RT) for high-reward/risk and low-reward/risk selections as well as the average RT across all selections.

### MRI protocol

#### Data acquisition

During the baseline visit, structural and functional images were acquired on a Siemens TIM Trio 3T scanner using a 12-channel head coil. During three runs of the WOF task, functional images were collected using a T2*-weighted gradient-echo planar imaging (EPI) sequence (interleaved slice acquisition, 47 axial slices per volume, TR = 2,500 ms, TE = 30 ms, TA = 2.48 ms, slice thickness = 3 mm, voxel size = 3.0 × 3.0 × 3.0 mm^3^, FoV = 192 × 192 mm^2^, flip angle = 90*^o^*).

High-resolution structural images were obtained using a T1-weighted magnetization prepared rapid acquisition gradient echo (MPRAGE) sequence (176 sagittal slices: TR/TE/TI = 920/2.52/900 ms, flip angle = 9*^o^*, slice thickness = 1.0 mm, FOV = 250 × 250 mm^2^, matrix of 256 × 256 for an effective spatial resolution of 0.97 × 0.97 × 1.0 mm^3^).

#### Functional MRI data pre-processing

Image pre-processing and statistical analyses were carried out using SPM8.^1^ Pre-processing included correction for interleaved slice timing, realignment of all images to the mean fMRI image to correct for head motion artifacts between images, and co-registration of realigned images to the anatomical MPRAGE. The MPRAGE was segmented and transformed into Montreal Neurological Institute (MNI) standard stereotactic space using non-linear warping. Lastly, these transformation parameters were applied to normalize the functional images into MNI space, and the data were spatially smoothed using a Gaussian kernel of 6 mm^3^ FWHM. A scrubbing algorithm utilizing frame-wise displacement was implemented to assess participant movement during the fMRI scans (92). Participants included in analyses demonstrated less than 1 mm displacement in fewer than 20% of their total volumes across all three runs of the task.

### Statistical analyses

#### Imaging data

First-level statistical analyses of imaging data included regressors encoding for trials during which the subject chose either the 10 or 30% probability (High-reward/risk) or the 70 or 90% probability (Low-reward/risk). Regressors of interest also included feedback trials on which subjects won (Win) or lost (Lose). Six translations and rotations modeling participant motion calculated during realignment were included as nuisance regressors.

Contrasts of interest examined whole brain activation for high-reward/risk compared to low-reward/risk trials (High-reward/risk > Low-reward/risk), and winning versus losing outcomes (Win > Lose). Regressors were convolved with the canonical hemodynamic response function. A temporal high-pass filter of 128 s was applied to the data to eliminate low-frequency noise (e.g., MRI signal drift). First-level contrasts of interest were used in a second-level analyses for comparisons between SI and SN groups. The initial cluster defining threshold was *p* < 0.001, with a cluster extent of 10 voxels (voxel size = 2.0 mm isotropic). Corrections for multiple comparisons were made using a cluster-level FWE threshold of *p* < 0.05. Macro-anatomical labels reported are based on peak coordinates and were assigned by the Harvard-Oxford Cortical/Subcortical Structural atlases (93–96), supplemented with labels from *Atlas of the Human Brain, 4*^th^* edition* (97).

#### Demographics and behavioral data

Statistical analyses were performed using R. Dependent variables were free from outliers and normality was examined. In the SN group, SES was negatively skewed (Shapiro-Wilk *W* = 0.88, *p* < 0.001). BAS fun-seeking scores were non-normally distributed in both groups (SI: *W* = 0.876, *p* < 0.05; SN: *W* = 0.934, *p* < 0.05); BIS was non-normal for SI (*W* = 0.897, *p* < 0.05) and BAS reward responsivity for SN (*W* = 0.923, *p* < 0.05). Further, the percent of high-reward/risk selections in the WOF task was positively skewed in both groups (SI: *W* = 0.757, *p* < 0.001; SN: *W* = 0.873, *p* < 0.001).

Standard transformations for the above dependent variables did not correct distributions; thus, the between-group comparisons were performed using non-parametric Mann-Whitney U test. Alpha was set at *p* = 0.05, and Bonferroni correction for multiple comparisons was applied where noted.

With the exception of the group comparisons for DUSI-R APD, all statistical tests were two-tailed. We used one-tailed tests in comparing groups on this measure given *a priori* evidence of directionality [i.e., DUSI-R APD severity, reflected by higher scores, positively predicts SU(74)].

## Results

### Demographics

Substance initiators and Substance non-initiators groups were similar in age, sex, PDS, SES, and race/ethnicity (Table 1). Mean IQ was not statistically different between SI and SN youth [*t*(68) = 1.92, *p* = 0.059]. However, we treated IQ as a covariate of no interest in imaging analyses given reported associations between cognitive ability and risk-taking (98–102), observed in our sample as well [percentage of high risk decisions was positively correlated with IQ (*r*_*s*_ = 0.28, *p* = 0.021; see Supplementary Material Section 1.2, Supplementary Figure 1, and Supplementary Table 2)]. Imaging results for analyses without IQ as a covariate are presented in the Supplementary materials (Supplementary Material Section 2.2, Supplementary Table 6, and Supplementary Figure 2).

**TABLE 1:** Participant characteristics at “baseline” assessment.

	All participants (*N* = 70)	SI (*n* = 27)	SN (*n* = 43)	Test statistic	*p*
**Age at scan** Mean (SD) Range	12.7 (0.66) 11.1–14.0	12.9 (0.61) 11.5–14.0	12.7 (0.69) 11.1–14.0	*t*(68) = −1.57	0.12
**Sex** Females, *n* (%) Males, *n* (%)	40 (57%) 30 (43%)	15 (56%) 12 (44%)	25 (58%) 18 (42%)	*χ^2^* = 0.045(1)	0.83
**PDS** Mean (SD) Females Males	2.3 (0.67) 2.58 (0.67) 1.94 (0.47)	2.4 (0.64) 2.72 (0.65) 2.1 (0.46)	2.2 (0.68) 2.49 (0.68) 1.83 (0.47)	*t*(68) = −1.42 *t*(38) = −1.07 *t*(28) = −1.55	0.16 0.29 0.13
**Race,** n (%) African American Caucasian Hispanic/Latino Other	19 (27%) 42 (60%) 4 (6%) 5 (7%)	6 (22.2%) 15 (55.6%) 3 (11.1%) 3 (11.1%)	13 (20.2%) 27 (62.8%) 1 (2.3%) 2 (4.7%)	*χ^2^*(3) = 3.75	0.29
**FSIQ** Mean (SD)	112.1 (15.3)	107.7 (14.4)	114.8 (15.4)	*t*(68) = 1.92	0.059
**SES Index *z*-score** Median (range) *Parental education, years, mean (SD)* *Household income, median*	0.22 (−2.7–1.45) 16.6 (2.8) 100,000–149,000	0.22 (−2.0–1.45) 16.5 (2.8) 100,000–149,000	0.26 (−2.68–1.36) 16.6 (2.8) 100,000–149,000	*U* = 599	0.82
**Family history**	10 (14.9%)	*n* = 26	*n* = 41	*χ^2^* = 4.82(1)	0.028*
(FH±), *n* (%)	57 (85.1%)				
*N* = 67,					
FHP		7 (26.9%)	3 (7.3%)		
FHN		19 (73.1%)	38 (92.7%)		

PDS, pubertal development scale; FSIQ, full-scale IQ; SES, socioeconomic status.

*Significant at *p* < 0.05.

Among SI adolescents (*n* = 27), 12 (44%) and 15 (56%) reported SU initiation at Wave 2 and Wave 3 follow-up, respectively. While different in age of initiation [Wave 2: *M* = 14.79, SD = 0.41; Wave 3: *M* = 15.78, SD = 0.70; *t*(25) = −4.3, *p* < 0.001], initiators at both time points were similar in demographic characteristics sex [χ^2^(1) = 1.08, *p* = 0.299]; race/ethnicity [χ^2^(1) = 4.72, *p* = 0.19]; age at initial assessment [*t*(25) = 0.50, *p* = 0.62]; IQ [*t*(25) = −1.69, *p* = 0.10]; pubertal development [*t*(25) = 1.66, *p* = 0.11]; SES [*t*(25) = −1.26, *p* = 0.22] (see Supplementary Materials Section 2.4 and Supplementary Tables 9–12, for SI group SU details). We therefore considered the SI initiators in aggregate, regardless of Wave.

The SI group had a higher proportion of FHP individuals. It is important to note that FHP youth who initiate use early are at particularly heightened risk for problematic SU (103); thus, it is possible that those FHP youth in the SI group may be at dramatically increased risk for SUDs relative to FHP youth in the SN group. Thus, in examining neural activation in SI and SN youth during reward-related decision making, we conducted *post-hoc* analyses that controlled for FH status (in addition to IQ). These results are reported in Supplementary Material Section 2.3, Supplementary Tables 7, 8, and Supplementary Figures 3, 4).

### Behavioral results

#### DUSI-R APD, BIS/BAS, and DD

A one-tailed independent samples *t*-test showed adolescents in the SI group had significantly higher scores on the DUSI-R APD compared to the SN group [*t*(67) = −1.89, *p* = 0.03] (Table 2), suggestive of increased problematic behavior in domains predictive of a future SUD. Compared to the SN group, SI adolescents had significantly higher scores on the BAS Drive [*t*(68) = −2.6, *p* = 0.012] and Fun Seeking (*U* = 362.5, *p* = 0.008) scales, but did not differ for BAS Reward Responsiveness or discounting behavior (Table 2) indicating similarities in aspects of reward processing.

**TABLE 2:** DUSI-R APD, DD, and BIS/BAS results.

	All participants (*N* = 70)	SI (*n* = 27)	SN (*n* = 43)	Test statistic	*p*
**DUSI-R APD** Mean (SD) *N* = *69*	16.04 (10.28)	*n* = 26 19 (11.5)	14.25 (9.1)	*t*(67) = −1.9	0.031*
**DD, AUClogd** Mean (SD) *N* = *61*	0.55 (0.19)	*n* = 25 0.54 (0.19)	*n* = 36 0.55 (0.19)	*t*(59) = 0.22	0.83
**BAS Drive** Mean (SD)	9.7 (2.7)	10.7 (2.4)	9.1 (2.7)	*t*(68) = −2.6	0.012^†^
**BAS Fun-seeking** Median (Range)	11 (4–15)	13 (6–15)	11 (4–15)	*U* = 362.5	0.008^†^
**BAS Reward Responsivity** Median (Range)	18 (14–20)	18 (14–20)	18 (14–20)	*U* = 516.5	0.44
**BIS** Median (Range)	20 (12–27)	20 (12–24)	18 (15–27)	*U* = 552.5	0.74

Group comparisons for the DUSI-R APD used a one-tailed independent samples *t*-test. Comparisons for AUClogd and the BAS Drive scale used two-tailed independent samples *t*-tests; and those for the BAS Fun Seeking and Reward Responsivity scales and the BIS were conducted using two-tailed Mann–Whitney *U*-tests. *p < 0.05; ^†^*p* < 0.0167. Bonferroni-corrected threshold for BAS sub-scales. DUSI-R APD, Drug Use Screening Inventory, Revised, Absolute Problem Density; DD, AUC, delay discounting, area under the curve; BAS, Behavioral Activation System; BIS, Behavioral Inhibition System.

### WOF task behavior

The groups made similar proportions of high-reward/risk selections (*Z* = 0.537, *p* = 0.70) (Table 3). A two-way repeated measures ANOVA was used to examine the effect of group (SI vs. SN) and selection type (high-reward/risk vs. low-reward/risk) on response time. A main effect of selection type was found, with both SI and SN groups taking significantly more time to make riskier selections compared to safer ones [*F*(1,68) = 65.7, *p* < 0.0001]. There was no significant main effect of group [*F*(1,68) = 0.0006, *p* = 0.98], nor was there a significant group × selection type interaction [*F*(1,68) = 0.007, *p* = 0.93].

**TABLE 3:** Wheel of Fortune task behavior: Descriptive statistics.

	All participants (*N* = 70)	SI (*n* = 27)	SN (*n* = 43)
**High-reward/risk selections, %** Median (range)	10 (1–59)	10 (1–59)	11 (1–55)
**High-reward/risk selections** RT (ms) Mean (SD)	1,240 (410)	1,240 (390)	1,240 (420)
**Low-reward/risk selections** RT (ms) Mean (SD)	990 (300)	990 (270)	990 (310)
**All selections** RT (ms) Mean (SD)	1,120 (330)	1,110 (320)	1,120 (350)

### Functional MRI results

**FIGURE 2:**
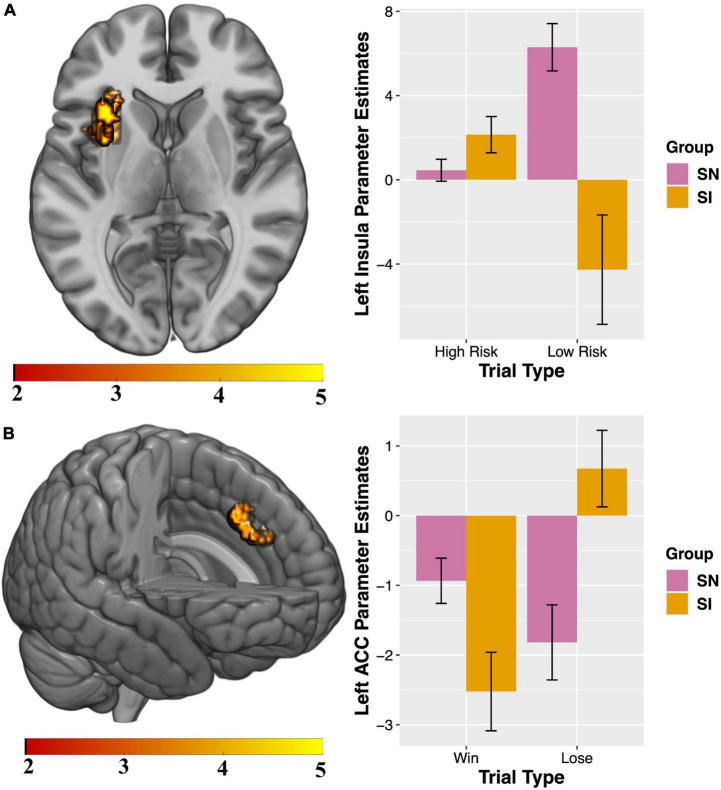
Between-group results for which substance non-initiators (SN) participants demonstrate increased activation relative to Substance Initiators (SI) adolescents. Interaction charts depict mean parameter estimates (error bars represent standard errors) for **(A)** High-reward/risk > Low-reward/risk, left insula; and **(B)** Win > Lose, left anterior cingulate cortex (ACC). FSIQ as covariate of no interest. Initial cluster defining threshold = *p* < 0.001, *k* = 10 voxels. Results survive FWE cluster-correction at *p* < 0.05.

Compared to SN youth, SI adolescents demonstrated less activation in the left insula when selecting high-reward/risk versus low-reward/risk options. Additionally, when presented with winning versus losing feedback, SI adolescents showed less activation in the left cingulate gyrus, relative to their SN peers (Table 4 and Figure 2). There were no regions for which SI youth showed greater activation compared to SN youth for either contrast (see Supplementary Materials Section 2.1 and Supplementary Table 5 for presentation of within-group fMRI results). Results surviving corrections for multiple comparisons are presented in Table 4 and Figure 2.

**TABLE 4:** Summary of SN > SI cluster-level corrected results for two contrasts of interest (IQ as covariate of no interest).

Region	Cluster size	MNI coordinates			*Z*	*t*	Corrected *P*-value (FWE)
		x	y	z			
**High-reward/risk > Low-reward/risk**							
Left insular cortex	492	−34	8	6	3.91	4.16	0.001
**Win > Lose**							
Left anterior cingulate gyrus	207	−4	20	38	4.22	4.54	0.031

Initial cluster defining threshold = *p* < 0.001, *k* = 10 voxels. Reported results survive FWE cluster-correction (*p* < 0.05).

### Exploratory analyses: *Post-hoc* tests of parameter estimates

Visual inspection of the parameter estimates in Figure 2 suggest that the significant between-group results for the contrasts of interest may be driven by differences in how the groups are processing each trial type during selection and feedback. To probe these potential differences, exploratory tests were conducted. All tests were two-tailed. Follow-up independent-samples *t*-tests showed groups differed significantly in activity during low risk [*t*(68) = 2.97, *p* = 0.004] but not high-risk [*t*(68) = −1.25, *p* = 0.22] trials (Figure 2A). Paired-samples *t*-tests examining within-group processing of high- compared to low-risk trials showed significant differences for SN [*t*(42) = −3.89, *p* = 0.0004] but not SI youth [*t*(26) = 1.95, *p* = 0.062]. Independent-samples *t*-tests showed groups significantly differed in brain response to losing [*t* = (68) = −2.17, *p* = 0.034] but not winning [*t*(68) = 1.84, *p* = 0.07] feedback (Figure 2B). Paired-samples *t*-tests revealed SI youth [*t*(26) = −4.35, *p* = 0.0002] significantly differed for activity when processing winning and losing trial types, while SN do not [*t*(42) = 1.59, *p* = 0.12]. Implications of these exploratory analyses as they relate to the between-group findings for High-risk > Low-risk and Win > Lose contrasts are treated in the Discussion.

## Discussion

This study aimed to characterize “baseline” behavioral and neural profiles of reward-sensitivity/risk-taking that distinguished SU-naïve early adolescents who did versus did not report SU initiation at 18- and 36-months follow-up. We sought to address an important gap in the literature—characterizing these behavioral and neural profiles in early adolescents *prior to substance exposure*. The elucidation of such profiles prior to substance initiation is critical, as the examination of behavioral/neural factors after SU initiation limits the ability to disentangle factors that may be antecedents of SU from those that may be a consequence of SU.

Our hypotheses concerning behavioral and neural profiles of SI and SN youth were partially confirmed. SI and SN adolescents significantly differed on self-report measures of reward-sensitivity/risk-taking, including the BAS Drive and Fun Seeking subscales, and the DUSI-APD. Unlike self-report measures, task-based metrics did not distinguish SI and SN youth, who demonstrated similar ability to delay gratification and similar risk-taking behavior. Despite similar risk-taking behavior, however, SI and SN differed for brain activity in in left-lateralized insula during risky decisions, and in the left anterior cingulate cortex (ACC) when presented with rewarded outcomes. Parameter estimates for contrasts of interest (i.e., High-Risk/Reward > Low-Risk/Reward, Win > Lose) indicated that groups showed distinctive patterns of activation for the different trial types (e.g., High-Risk/Reward, Low-Risk/Reward), a finding that, to our knowledge, is novel, and highlights the importance of conducting neuroimaging to examine neural responses contributing to behavior.

### Behavioral profiles of reward-sensitivity/risk-taking

We predicted that SI youth would show greater sensitivity to potential rewards and lower aversion to potential punishments reflected by higher BAS and lower BIS scores. These hypotheses were partially confirmed. Groups did not differ for the BAS Reward Responsivity scale or the BIS. However, SI youth showed higher scores on the BAS Drive and Fun-Seeking scales. Elevated scores on these two BAS scales have been associated with low levels of harm avoidance (85) and problematic drug or alcohol use (104, 105), including in adolescents (106–110). Further, these two scales were positively correlated with adolescent risky choices during a win-only (but not a lose-only) version of a WOF task (111). Thus, elevated scores on these BAS scales prior to SU may reflect a propensity toward affective and behavioral responsivity to rewarding stimuli in SI youth, potentially biasing these individuals toward greater risk-taking and decreased harm avoidance (112).

In contrast to the literature on the BAS, findings concerning associations between BIS and SU have been less consistent. BIS was negatively correlated with SU among adolescents (escalation of cannabis use) (113) and college students (amount and frequency of alcohol consumption) (114). However, BIS scores of adolescents aged 15–18 years at baseline did not predict substance misuse 2 years later (52), findings consistent with the current study.

Compared to their SN peers, SI adolescents also showed elevated DUSI-R absolute problem density (APD) scores. The APD score is a multi-dimensional construct, quantifying adolescent difficulties across health, psychosocial and psychiatric domains associated with SUD (73). As such, elevated APD scores in SI adolescents may reflect increased relative risk for SU initiation. Importantly, however, 69% of SI youth would not be considered high risk according to the previously established cut-off score of 24 (74). Further, similar proportions of adolescents in each group scored ≥24 [SI: 30% (8/27); SN: 19% (8/43); χ*^2^* = 0.70(1), *p* = 0.40], highlighting the necessity of considering APD scores as a component of a more comprehensive risk profile.

We predicted that at baseline, those who would go on to initiate SU at 18- or 36-months follow-up would overvalue immediate gratification on a delay discounting task. This hypothesis was not confirmed. SI and SN youth did not differ on performance on the DD task, suggesting similar preference for immediate rewards under current task parameters. Unlike the BIS/BAS and DUSI-R, which query real-world preference and situationally-based behavior, the laboratory DD task (like WOF) may lack the sensitivity to detect group differences *prior to SU initiation* (115).

We further predicted that those who went on to initiate substances would make riskier choices in a reward-related decision-making task compared to adolescents who remained SU-naïve throughout the study. However, we found that task-based risk-taking was similar between groups, and across all participants selection of high-reward/risk options was accompanied by longer deliberation than low-reward/risk options, an effect consistent with previous studies (67, 89, 111, 116).

### Neural profiles of reward-sensitivity/risk-taking

We hypothesized that SI and SN youth would show baseline differences in brain activity during the WOF task in regions associated with decision-making under uncertainty and in reward processing/sensitivity, and more specifically, the medial prefrontal cortex and ventral striatum and amygdalae, respectively. SI and SN youth did not show differences in ventral striatum or amygdalae, as predicted; however, SI and SN groups differed in neural activity underlying *making* risky selections and *processing* rewarded outcomes despite similar task-based behavior.

SN adolescents demonstrated significantly greater left insular cortex response to High-Reward/Risk versus Low-Reward/Risk trials (Figure 2A). *Post-hoc* exploratory analyses indicated that during risk-taking SI and SN groups differed in patterns of activation depending on whether they chose a high- or low-risk option, suggesting that the marked difference in responsivity to Low-Reward/Risk trials drives the significant between-group result for the High-reward/risk > Low-reward/risk contrast. Within-group exploratory analyses revealed that only SN adolescents showed significantly different activation for High-Reward/Risk compared to Low-Reward/Risk trials, while brain response to the two trial types was similar within the SI group.

During decision-making, the insular cortex plays a role in refocusing attention based on salience, evaluating risk, inhibiting action, and processing outcomes (112, 117–119). Attenuated insula activity is associated with increased real-world risk-taking among adolescents (120, 121), and aberrant insula engagement in processing salient stimuli is observed in individuals with addiction (122–124). Reduced activation of the anterior insula has been found to play an important role in adolescent risky decision making in comparison to adults and is linked to more emotionally driven decisions (125). Taken together, our results may be indicative of relative immaturity in the SI group in a region that plays an important role in evaluating degree of risk (126), potentially reflecting a neuroendophenotypic vulnerability to the early initiation of substances of abuse in these adolescents. SN youth showed greater left ACC response to winning outcomes. Parameter plots suggested that while processing outcomes related to gain or loss, SN and SI adolescents demonstrated differing patterns of responses in this region (Figure 2B). *Post-hoc* exploratory analyses revealed groups significantly differed for activation during losing, but not winning, feedback. Further, only SI youth showed significantly different activation for Lose compared to Win trials. Increased ACC activity has previously been associated with processing gains (relative to no gains) in a gambling task in adolescents (116). Individuals with established SUDs show impairments in decision-making (127), altered ACC structure (128) and differences in brain activity during risk-taking (129). Specifically, individuals with SUDs display not only greater substance-related cue-induced ACC activity during active use (130), but also blunted ACC activity during decision-making while abstinent (131), an effect which predicts craving, length of time to relapse and relapse severity (132). Importantly, some of these differences may be evident prior to development of AUD/SUD including alterations in ACC neuroanatomy (133) and these differences may reflect increased vulnerability to SUDs (e.g., youth with positive family history of alcoholism demonstrate hyper-activation during risk-taking compared to youth with negative family history) (134).

Both the insula and ACC are implicated in reward-related decision-making (66, 89, 120, 126, 135, 136), and as hubs in the salience network, the anterior insula, and ACC (137, 138) integrate automatic, bottom-up detection of relevant internal and external stimuli with cognitive, top-down processing (139). The salience network is implicated not only in altered cue-reactivity among individuals with SUD (122, 123), but may play a contributory etiological role in early SU and transition to SUD (140). While others have established that adults with SUD demonstrate aberrant patterns of insular and cingulate activity during risky decision-making (141) and that reduced insular activity during risk-related processing is predictive of relapse (142), our results suggest that variability in insular and ACC activity is present in individuals at risk for SUD *prior to* substance exposure.

The exploratory results intriguingly suggest SI youth may be more neurally sensitive to the distinction between wins and losses, though this remains to be empirically tested in planned comparisons correcting for multiple comparisons. It is also possible that steep hypoactivation of the ACC in SI adolescents in the context of rewarding outcomes indicates an increased threshold for rewarding stimuli (consistent with elevated BAS fun-seeking scores SI youth). These group differences may reflect differences in outcome monitoring and processing (143) and awareness of outcomes (144), which serve in part to guide behavior (145, 146). A notable consistency between present findings and previous studies is that youth with differential risk for SU demonstrate similar task performance but differences in patterns of brain activation across a variety of tasks (147, 148). Thus, early disruptions in PFC function, including ACC, may contribute to a constellation of impairments including aberrant response inhibition and salience assignment (149) and ultimately to real-world risky decision making.

### Strengths and limitations

An important strength of the current study is the stringent inclusion criteria implemented to ensure SU-naïve status of youth at initial assessment, and the requirement of convergent responses on two SU measures (DUSI-R and TAD) to classify youth at follow-up. In contrast, previous studies examining “SU-naïve” youth include those who report “little to no” alcohol use (150), or who do not report “significant” (65, 69, 151) or “heavy” alcohol or drug use (152). Others rely on urine drug screening at scanning time (91), which, for many drugs, capture only recent use (153) and are not reflective of patterns of use over time.

Another strength of the current study is the narrow age range at baseline (11–13-year-olds). While previous studies enrolled participants with a more distributed age range (66, 91, 151), we restricted eligibility at enrollment to a much smaller range in an effort to capture information regarding early initiation and to minimize potential age-related confounds in neurodevelopment. Finally, the current sample of adolescents were well-characterized using a battery capturing a variety of factors presumed to confer risk for or resilience to early SU, including preference for immediate gratification (DD), affective and behavioral responsivity to rewards and punishment (BIS/BAS), multidimensional risk for SU problems (DUSI-R). Additionally, follow-up neuroimaging analyses controlled for an important factor associated with SUD risk (family history status) and results were largely similar to those reported in the main analyses.

On the other hand, by analyzing the selection phase of the WOF in a version of the task that consistently coupled high reward with low probability and low reward with high probability we were unable to dissociate between patterns of activation associated with reward versus risk. Although this limitation is not unique to the current study (154), it is unclear here whether between-group differences in insular cortex were driven by reward sensitivity or risky decision-making. Future inclusion of choices with equal probability of high/low reward (i.e., 50/50 wheels), as in the “classic” WOF task, will permit testing the relative contributions of reward magnitude independent of perceived risk (67). It is important to note, however, that estimation of reward value and tendency toward risk outside of the laboratory may not be not entirely separable either; decisions with greater reward potential, whether adaptive (e.g., approaching a classmate to initiate a conversation) or maladaptive (e.g., underage alcohol consumption) are inherently accompanied by risk (e.g., social consequences such as peer rejection; or adverse physiological impacts of alcohol consumption and parental or school punishment for drinking).

A notable strength of the current study was its implementation of stringent criteria to ensure the SU-naïve status of youth at baseline, with the exception of nicotine use which was not exclusionary. Given associations of nicotine exposure in adolescence with alterations in brain development (155) and given associations of nicotine use with initiation and use of other substances (156, 157), the inclusion of participants who reported past nicotine use during the initial study visit reflects a limitation of the current study. Mitigating impacts of this limitation on the study’s findings, however, is that only two of the 70 participants reported nicotine use at baseline, and one participant went on to initiate other substances while one of these participants did not.

The current study identified youth who initiated at different ages (initiation at approximately 18- vs. 36-months follow-up), which may also limit the interpretation of our outcomes. Although earlier and later initiators were similar in demographic, physical, and cognitive characteristics, as well as task-based behavior and BIS/BAS scores, those who reported earlier initiation (approximately 18-month follow-up) scored higher on the DUSI-APD, indicative of greater risk in domains that precede or co-occur with problematic SU. Due to concerns regarding statistical power, we were unable to compare SI subgroups on brain activation during the WOF task. Future studies should recruit greater numbers of participants who are likely to be assigned to one of these two SU subgroups to prospectively examine group differences in neural activation among individuals who initiate at different stages of adolescence.

Relatedly, the current study is unable to determine pathways to SU escalation, and SUD. SU initiation itself, while necessary, is not sufficient to promote continued or escalated use or the eventual entrenchment of pathways that might be specific to SUD risk. The elucidation of factors that give rise to such pathways, including early brain biomarkers, may provide a much richer understanding of how brain functioning in SU-naïve adolescents portends subsequent life course outcomes.

### Implications and directions for future research

Overall, our findings are consistent with the premise that differences in regional PFC activity may occur *prior* to SU initiation and thus may confer vulnerability to SUDs (149, 158). A novel finding indicates that *variability in activity* in ACC and insula—key regions known to support reward- and risk-related decision-making—may distinguish SU-naïve early adolescents who initiate SU earlier from those who remain abstinent. The findings reported here furthermore lend support to models suggesting that divergent neurodevelopmental trajectories may be precede SU, and point to the potential promise of developing interventions to target these key brain regions and the behavioral functions they support before SU initiation to disrupt maladaptive and/or promote more adaptive trajectories (159).

## Data availability statement

The data analyzed for this study are not readily available due to the inclusion of sensitive material about adolescent participants, including linkages between background and substance use. Requests to access de-identified datasets should be directed to dfishbein@unc.edu.

## Ethics statement

This study was approved by the Georgetown University Institutional Review Board. Written informed consent and assent were obtained from the parent and adolescent, respectively.

## Author contributions

DHF and ASV contributed to the conceptualization and design of the study. GM and AP performed statistical analysis. GM wrote the first draft of the full manuscript. AP assisted in the preparation of the initial draft of the methods and organized the dataset. GM, VLD, EJR, DHF, and ASV contributed to manuscript revisions. All authors read and approved the submitted version.

## Footnotes

1
http://www.fil.ion.ucl.ac.uk/spm
