# Supplementary material for: Baseline brain and behavioral factors distinguish adolescent substance initiators and non-initiators at follow-up
Source: Front Psychiatry. 2022 Dec 8;13:1025259. doi: 10.3389/fpsyt.2022.1025259 (PMC9780121; doi:10.3389/fpsyt.2022.1025259)
Supplement: Supplementary file 1 [file Data_Sheet_1.docx]

Supplementary Material

# 1. Supplementary Methods

**Supplementary Table 1.** Participants remaining after each stage of data filtering based on inclusionary/exclusionary criteria

|  | *N* |
| --- | --- |
| 1. **Enrolled in study** | **135** |
| 2. **Neurodevelopmental disorder** | 1 |
| 3. **Indeterminate use status** | **21** |
| 4. **Excluded due to incomplete or insufficient imaging or task data**   1. No imaging data 2. Technical problems during imaging data acquisition 3. Incomplete imaging data 4. No high-reward/risk selections during task | 1  10  4  4 |
| 5. **Excluded due to excessive head motion** | **24** |
| TOTAL excluded | 65 |
| **Final sample** | **70** |

*1.1. Wheel of Fortune (WOF) task*

During each trial of a modified WOF task(1), participants were presented with a ‘wheel’ (a probability pie-chart), divided into two unequal slices, which depicted the odds of winning hypothetical monetary rewards associated with those slices (Figure 1). Across 90 trials (3 x 30 trial runs; approximately 21 minutes in total), the odds of winning/losing were randomly varied between 10% vs. 90% (32-42 trials) and 30% vs. 70% (48-58 trials). Monetary amounts ranged from $1 to $21. During the selection phase of the task, adolescents chose between a relatively small chance (10% or 30%) of winning/losing a large reward ($9, $18 or $7, $21) (i.e., ‘high-reward/risk’ selection), versus a greater chance (90% or 70%) of winning/losing a smaller reward ($1, $2 or $3, $9) (i.e., ‘low-reward/risk’ selection).

The present study used a version of the WOF task that was modified to reduce scan time and minimize fatigue for our early adolescent participants. This included excluding trials with equal odds (50/50) of winning either value and the anticipatory phase, during which the participant rated their confidence in their selection outcome. Further, the task was modified so that both ‘wins’ *and* ‘losses’ were possible outcomes.

Participants made a selection by pressing the button on the side corresponding to the color of their choice (Figure 1). Participants were given 3000ms to make a selection, and failure to respond within the allotted time resulted in loss of the highest dollar value offered for that trial. Selections were followed by a 3000 ms delay preceding the feedback phase during which participants viewed a screen informing them of the trial outcome along with a running total of their winnings.

*1.2. BIS/BAS*

The BIS/BAS is a 20-item self-report measure answered on a 4-point Likert scale (“Very true for me,” “Somewhat true for me,” “Somewhat false for me,” “Very false for me”)(2). The BIS is a single, 7-question scale that probes behavioral and emotional responsivity to punishment). The BAS, on the other hand, is comprised of 3 subscales: Reward Responsiveness (5 questions, which probe affective responsivity to rewards), Drive (4 questions, which probe persistence in the pursuit of rewards), and Fun Seeking (4 questions, which probe a desire for novel rewards and spontaneity in acting to obtain rewards). A higher BIS score reflects aversion to and avoidance of potential punishment; while higher BAS subscale scores reflect positive emotionality (Reward Responsiveness) and behavioral approach (Drive and Fun Seeking) in the context of potential rewards.

*1.3 Associations between WOF task behavior and full-scale IQ (FSIQ)*

We examined whether FSIQ was associated with the percent of high-reward/risk decisions and RT for high-reward/risk decisions in WOF task behavior. In the total group (*N*=70) FSIQ was positively correlated with the percent of high risk decisions for all three runs of the task (*r*_s_ =.28, *p*=.021) (Supplementary Figure 1A). Looking more closely at the percent of high-reward/risk decisions by run (Supplementary Table 2) we found that this correlation is driven by the association of FSIQ with high-reward/risk decisions in the first run of the task (*r*_s_=.31, *p*=.0083) (Supplementary Figure 1B). There were no significant associations between FSIQ and either the second (*r*_s_ =.09, *p*=.44) or third run of the task (*r*_s_ =.04, *p*=.74). Using the R package WRS2, a robust repeated-measures mixed ANOVA showed only a main effect for Run (*F*(2,28)=7.99, *p*=.002), with no significant effect for group (*F*(1, 32)=.17, *p*=.68) or the run × group interaction (*F*(2,28)=.14, *p*=.87) (Supplementary Table 2). Mean RT for high-reward/risk decisions was not significantly correlated with FSIQ, neither for all 3 runs nor for any single run (all *p*s >.05).

**Supplementary Table 2**. Percent of High-reward/risk selections on the WOF task by run

|  | SI  *n*=27 | SN  *n*=43 |
| --- | --- | --- |
| **High-reward/Risk selections, Run 1, %**  Mean(SD)  Median(range) | 21.8(21.7)  13.3(0-76.7) | 22.3(20.6)  16.7(0-72.4) |
| **High-reward/Risk selections, Run 2, %**  Mean(SD)  Median(range) | 15.5(15.9)  10(0-53.3) | 15.5(16.6)  10(0-60) |
| **High-reward/Risk selections, Run 3, %**  Mean(SD)  Median(range) | 10.8(15.0)  3.6(0-53.3) | 12.9(16.3)  6.7(0-60.7) |

**Supplementary Figure 1.** Scatterplots showing the association between FSIQ in total group (*N*=70) with the percent of high-reward/risk (HR) decisions A) for all three runs of the WOF task and B) for the first run of the WOF task.

**B**

**A**

**Supplementary Table 3**. Number of participants with zero high-reward/risk selections by run

|  | SI  *n*=27 | SN  *n*=43 |
| --- | --- | --- |
| **No High-reward/risk selections, Run 1**  n(%) | 1(3.7) | 5(11.6) |
| **No High-reward/risk selections, Run 2**  n(%) | 3(11.1) | 6(13.9) |
| **No High-reward/risk selections, Run 3**  n(%) | 8(29.7) | 6(13.9) |

**Supplementary Table 4**. Mean response time (RT) for high-reward/risk selections by run

|  | SI  *n*=27 | SN  *n*=43 |
| --- | --- | --- |
| **High-reward/risk selections RT (ms), Run 1**  Mean(SD)  Median(range) | *n*=26  1.31(.45)  1.27(.55-2.39) | *n*=38  1.26(.43)  1.29(.33-1.95) |
| **High-reward/risk selections RT (ms), Run 2**  Mean(SD)  Median(range) | *n*=24  1.14(.31)  1.19(.61-1.7) | *n*=37  1.3(.51)  1.33(.28-2.25) |
| **High-reward/risk selections RT (ms), Run 3**  Mean(SD)  Median(range) | *n*=19  1.18(.37)  1.21(.59-1.98) | *n*=37  1.22(.49)  1.17(.3-2.43) |

# 2. Supplementary Results

*2.1 FMRI results: Within-group analyses*

In the selection phase (High-reward/risk > Low-reward/risk), the SN group demonstrated significant activation in the left insula. During the feedback phase (Win > Lose), SN adolescents demonstrated significant activation in the left putamen and superior frontal gyrus/middle frontal gyrus, and right precentral gyrus and cingulate gyrus (Supplementary Table 5). Among adolescents reporting SU initiation (SI group), no results survived correction for multiple comparisons for either contrast of interest (High-reward/risk > Low-reward/risk or Win > Lose).

**Supplementary Table 5.** Results for SN group for selection (High-reward/risk > Low-reward/risk) and feedback (Win > Lose) phases of WOF task. Initial cluster defining threshold=*p*<0.001, *k*=10 voxels. Reported results survive FWE cluster-correction (*p*<.05).

| Region | BA | Cluster size | MNI coordinates  x y z | | | *Z* | *t* | Corrected  *p*-value  (FWE) |
| --- | --- | --- | --- | --- | --- | --- | --- | --- |
| High-reward/risk > Low-reward/risk | | | | | | | | |
| L insular cortex | -- | 342 | -28 | 16 | -12 | 4.30 | 4.86 | .002 |
| Win > Lose | | | | | | | | |
| L putamen | -- | 1660 | -14 | 4 | -12 | 4.67 | 5.41 | .000 |
| R superior frontal gyrus | 9 | 508 | 20 | 38 | 48 | 4.54 | 5.21 | .000 |
| R precentral gyrus | 4 | 1091 | 2 | -22 | 72 | 4.53 | 5.20 | .000 |
| R cingulate gyrus | -- | 200 | 28 | 0 | 24 | 4.49 | 5.14 | .037 |
| L superior frontal gyrus/middle frontal gyrus | 8 | 671 | -24 | 34 | 50 | 4.48 | 5.12 | .000 |

*2.2 FMRI Results: Between-group analyses without FSIQ as a covariate of no interest*

**Supplementary Table 6.** Summary of SN > SI cluster-level corrected results for Win > Lose contrast (without FSIQ as a covariate of no interest). Initial cluster defining threshold=*p*<0.001, *k*=10 voxels, FWE cluster-corrected at *p*<.05.

| **Region** | **Cluster size** | **MNI coordinates**  **x y z** | | | ***Z*** | **Corrected *p*-value** |
| --- | --- | --- | --- | --- | --- | --- |
| Right cingulate gyrus | 423 | 2 | 16 | 24 | 4.81 | .001 |


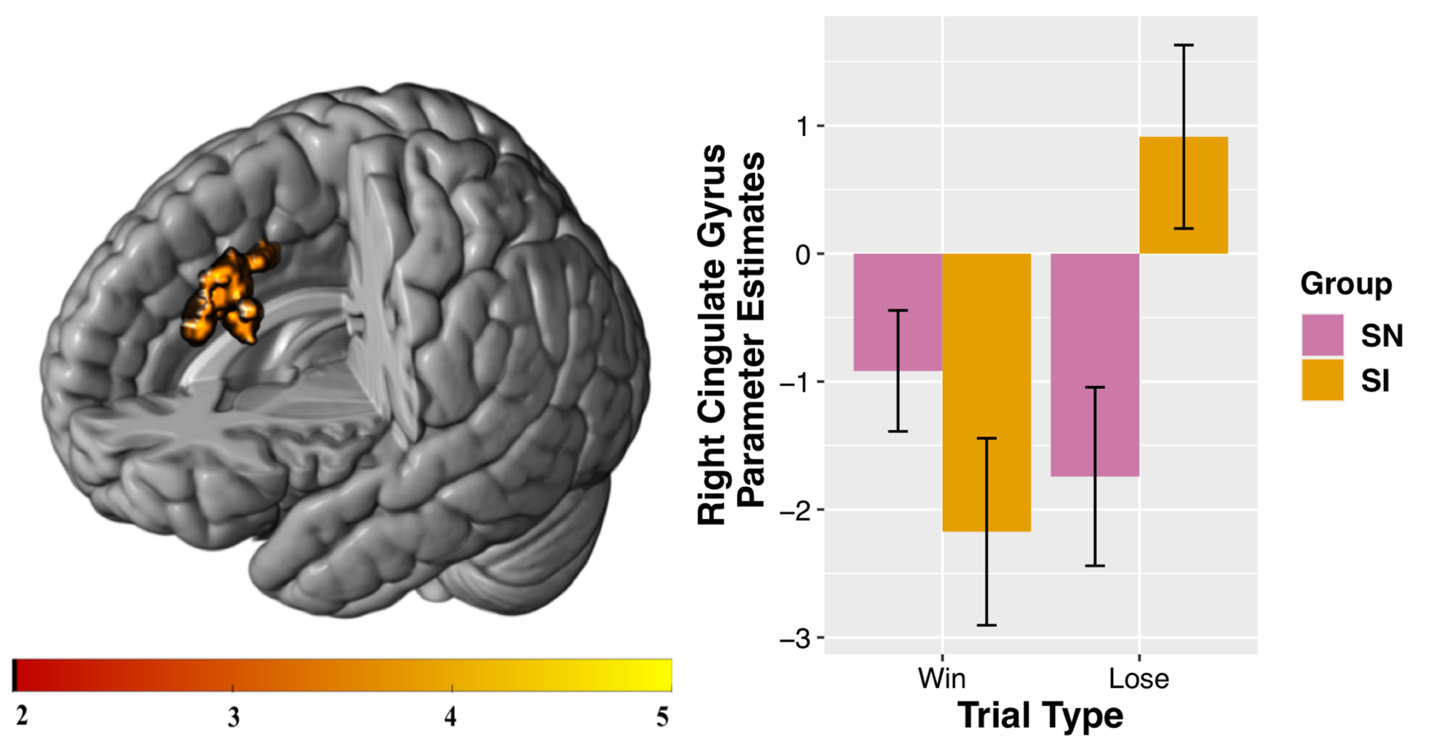


**Supplementary Figure 2.** Between-group results without FSIQ as a covariate of no interest. SN participants demonstrate greater activation relative to SI adolescents. Interaction charts depict mean parameter estimates and standard error for Win > Lose. Initial cluster defining threshold=*p*<0.001, k=10 voxels. Results survive FWE cluster-correction at *p*<.05.

*2.3 FMRI Results: Between-group analyses with FH status and FSIQ as covariates of no interest*

Because SI groups had a significantly greater proportion of FHP status youth relative to the SN group (see Table 1), imaging analyses were rerun to include FH status (along with FSIQ) as a covariate of no interest. Three participants without information concerning FH status (SI=1; SN=2) were dropped from analyses, leaving a sample of 67 (SI=26; SN=41).

For the contrast High-reward/risk > Low-reward risk, after controlling for both FH status and FSIQ, the results reported in the main text (in which only FSIQ was a covariate of no interest) remain unchanged: SN youth demonstrated greater activation in left insular cortex relative to SI youth, and the results survived FWE-correction for multiple comparisons (Supplementary Table 7, Supplementary Figure 3).

**Supplementary Table 7.** Summary of SN > SI cluster-level corrected results for HR > LR contrast with FH status and FSIQ as covariates of no interest. Initial cluster defining threshold=*p*<0.001, *k*=10 voxels, FWE cluster-corrected at *p*<.05.

| **Region** | **Cluster size** | **MNI coordinates**  **x y z** | | | ***Z*** | | ***t*** | | **Corrected**  ***p*-value (FWE)** | |
| --- | --- | --- | --- | --- | --- | --- | --- | --- | --- | --- |
| **High-reward/risk > Low-reward/risk** | | | | | | | | | | |
| Left insular cortex | 318 | -38 | 8 | -6 | | 3.92 | | 4.19 | | .01 |


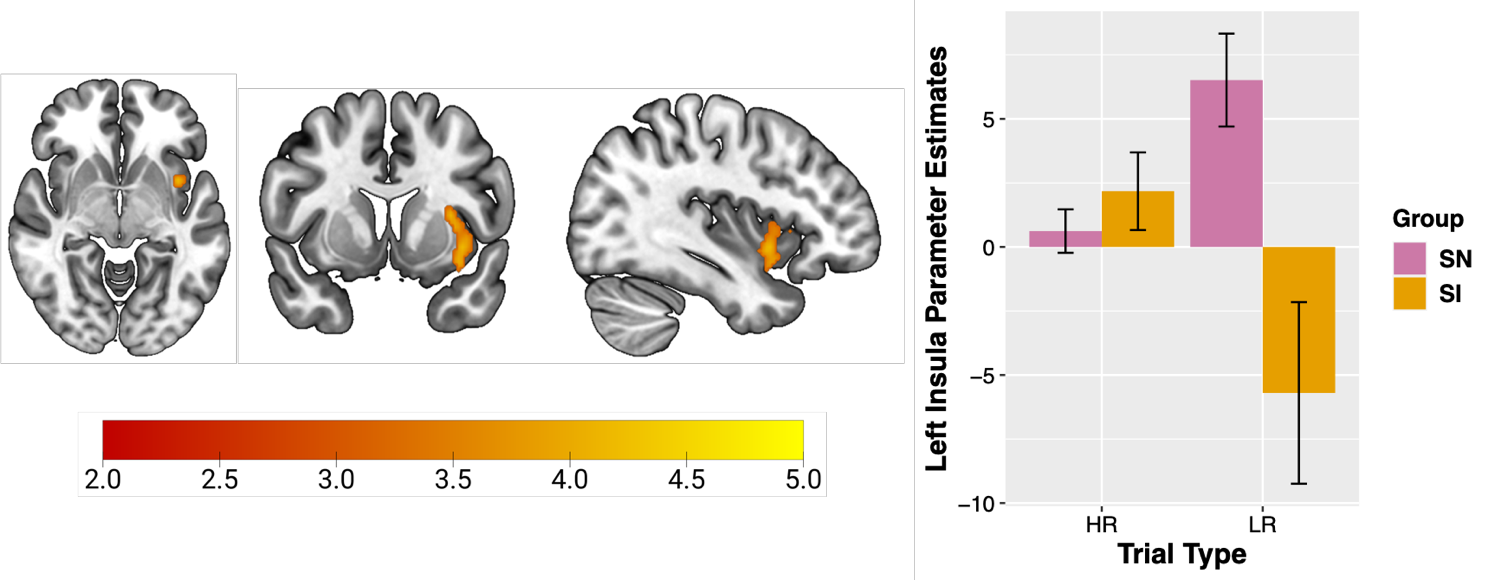


**Supplementary Figure 3.** Between-group results with FH status and FSIQ as covariates of no interest. SN participants demonstrate greater activation relative to SI adolescents. Interaction charts depict mean parameter estimates and standard error for HR > LR. Initial cluster defining threshold=*p*<0.001, k=10 voxels. Results survive FWE cluster-correction at *p*<.05.

For the contrast Win > Lose, after controlling for both FH status and FSIQ, SN youth demonstrated greater activation in regions proximal to those reported in the main text, and specifically regions of anterior cingulate cortex (Supplementary Table 8, Supplementary Figure 3). Unlike results reported in the main text (which controlled for only FSIQ), the results here did not survive corrections for multiple comparisons, likely due to reduced statistical power (due to the removal of *n*=3 without available FH status, and reduced degrees of freedom from an additional covariate). Importantly, however, the directionality of group differences for this contrast and the brain regions identified are consistent across the analyses in the main text and those reported here.

**Supplementary Table 8.** Summary of SN > SI for Win > Lose with FH status and FSIQ as covariates of no interest. Initial cluster defining threshold=*p*<0.001, *k*=10 voxels. Uncorrected as well as FWE-corrected p-values are reported. Results do not survive FWE cluster-correction at *p*<.05.

| **Region** | **Cluster size** | **MNI coordinates**  **x y z** | | | ***Z*** | ***t*** | ***p*-value**  **(uncorr.)** | ***p*-value (FWE)** |
| --- | --- | --- | --- | --- | --- | --- | --- | --- |
| **Win > Lose** | | | | | | | | |
| Left paracingulate gyrus/  anterior cingulate gyrus | 161 | -10 | 32 | 30 | 4.03 | 4.32 | .01 | .07 |
| Right anterior cingulate gyrus | 163 | 4 | 28 | 18 | 3.91 | 4.17 | .01 | .07 |


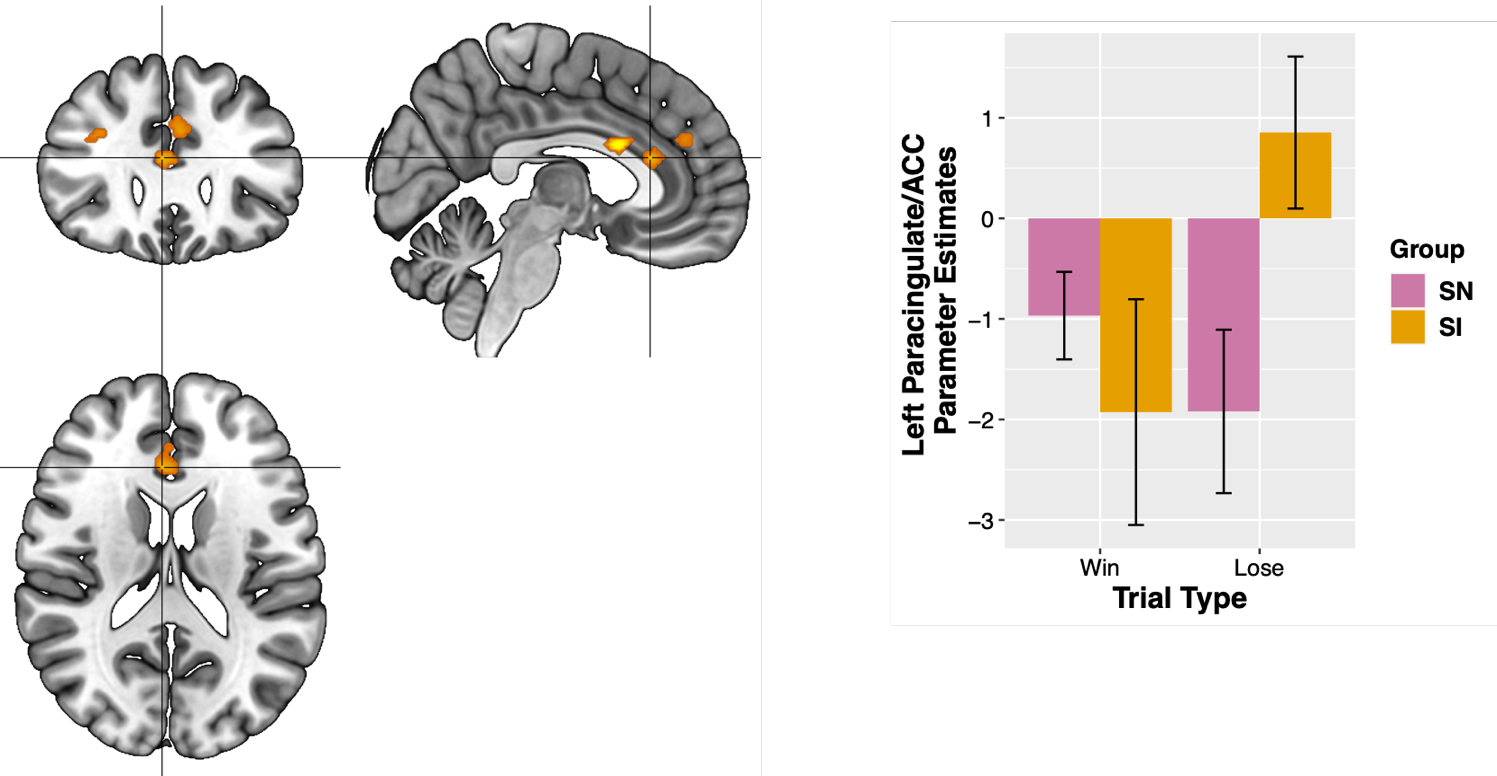


**A**


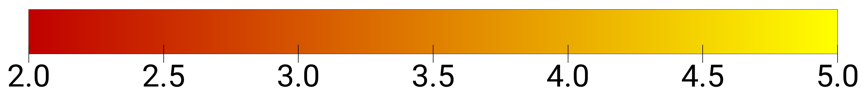


**B**


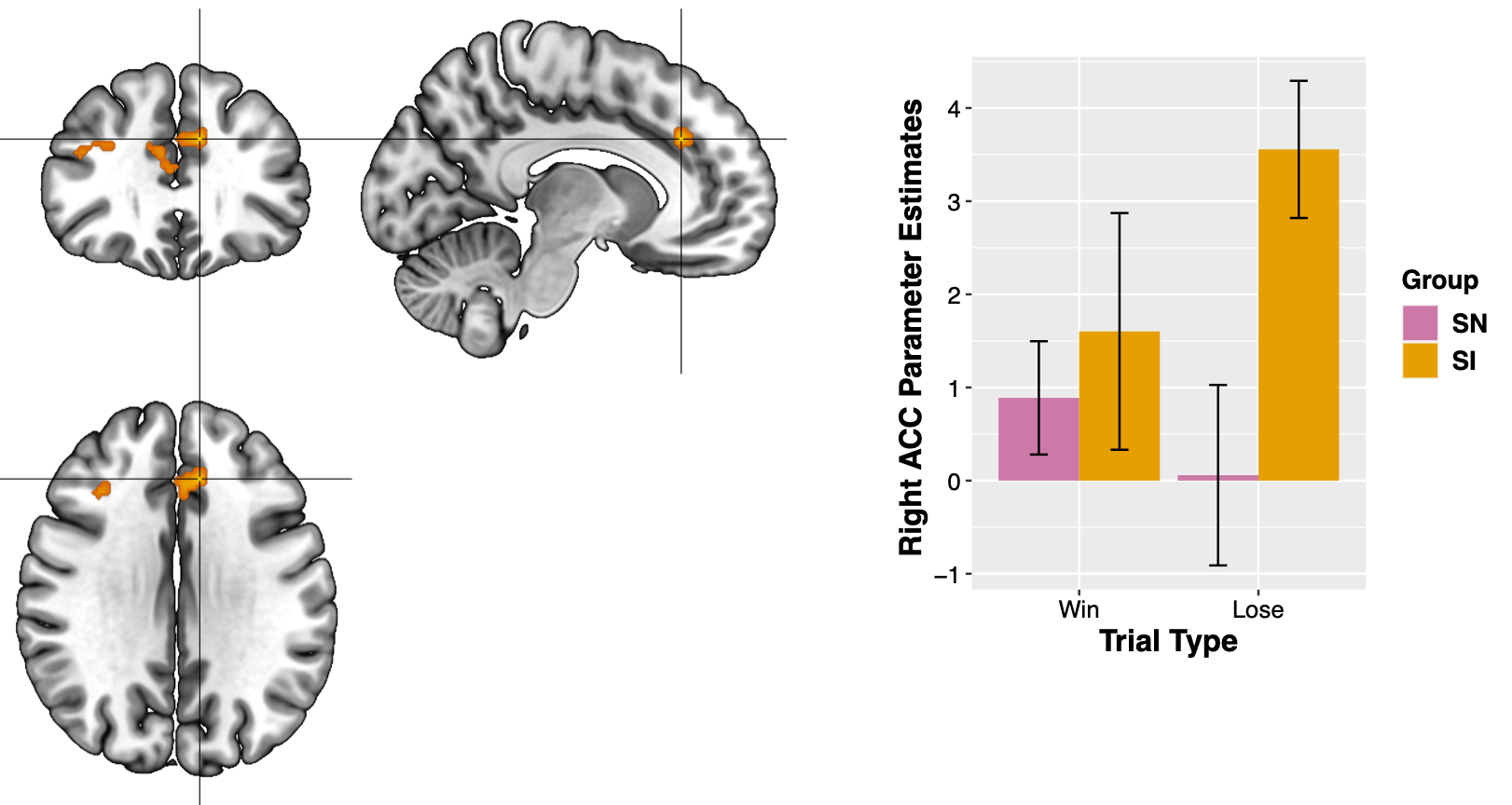


**Supplementary Figure 4.** Between-group results with FH status and FSIQ as covariates of no interest. SN participants demonstrate greater activation relative to SI adolescents. Interaction charts depict mean parameter estimates and standard error for Win > Lose. Initial cluster defining threshold=*p*<0.001, k=10 voxels. Results do not survive FWE cluster-correction at *p*<.05. Crosshair coordinates at -10, 32, 30 (A) and 4, 28, 18 (B).

*2.5 Characteristics of Wave 2 and Wave 3 SI participants*

**Supplementary Table 9**. Substance use reported by Wave 2 and Wave 3 SI participants

|  | **Wave 2 Initiators**  **(~18-months follow-up)**  (N = 12) | **Wave 3 Initiators**  **(~36-months follow-up)**  (N = 15) |
| --- | --- | --- |
| **Mean age at initiation** | 14.79 (0.41) | 15.78 (0.70) |
| **Substances reported** | Alcohol (11), marijuana (8), synthetic marijuana (4), spice (3), OTC (3), prescription painkillers (2), smoking tobacco (3), chewing tobacco (2) | Alcohol (13), marijuana (6), smoking tobacco (1), OTC (1) |
| **Polysubstance Users** | 9 | 4 |

OTC = over-the-counter medications used to get high.

**Supplementary Table 10.** Characteristics of Wave 2 and Wave 3 SI participants

|  | **SI (Wave 2)**  *n* = 12 | **SI (Wave 3)**  *n* = 15 | **Test statistic** | ***p*** |
| --- | --- | --- | --- | --- |
| **Age at scan**  Mean(SD) | 12.98(.57) | 12.86(.65) | *t*(25) = .50 | .62 |
| **Sex**  Females, n(%)  Males, n(%) | 8(67%)  4(33%) | 7(47%)  8(53%) | *χ^2^*(1) = 1.1 | .30 |
| **PDS**  Mean(SD)  Females  Males | 2.7(.59)  2.9(.47)  2.2(.59) | 2.3(.64)  2.5(.80)  2.1(.41) | *t*(25) = 1.7  *t*(13) = 1.2  *t*(10) = .52 | .11  .27  .61 |
| **Race, n(%)**  African American  Caucasian  Hispanic/Latina/o  Other | 3(25%)  5(42% )  1(8%)  3(25%) | 3(20%)  10(67%)  2(13%)  0(0%) | *χ^2^*(3) = 4.7 | .19 |
| **FSIQ**  Mean(SD) | 102.7 (14.2) | 111.7(13.6) | *t*(25) = -1.7 | .10 |
| **SES Index *z*-score**  Mean(SD)  *Parental education,*  *years, Mean (SD)*  *Household income,*  *Median* | -.25(1.15)  16.0(3.4)  $50,000 - $74,999 | .20(.71)  16.8(2.3)  $1000,000 - $149,999 | *t*(25) = -1.3 | .22 |
| **Family History**  (FH+/-), n (%)  *N* = 26  FHP  FHN | N = 12  4(33%)  8(67%) | N = 14  3(21%)  11(79%) | *χ^2^*(1)=.47 | .50 |

PDS = pubertal development scale, FSIQ = full-scale IQ, SES = socioeconomic, FHP = positive family history of alcohol/drug abuse, FHN = negative family history of alcohol/drug abuse.

*2.6 DUSI-R APD, DD task, and BIS/BAS: Wave 2 vs. Wave 3 SI participants*

Within the SI group, a one-tailed independent samples *t-*test of initial assessment/baseline DUSI APD revealed Wave 2 SI participants (mean: 23.15(12.59)) showed significantly higher scores compared to Wave 3 SI adolescents (mean: 15.44(9.55)) (*t*(24) = 1.78, *p* = .04)). Wave 2 and Wave 3 SI adolescents did not differ for DD, or for BIS/BAS subscales. See Supplementary Table 11.

**Supplementary Table 11.** DUSI-R, DD, and BIS/BAS subscales for Wave 2 and Wave 3 SI participants

|  | **All SI**  **N = 27** | **SI**  **(Wave 2)**  **N = 12** | **SI**  **(Wave 3)**  **N = 15** | **Test statistic** | ***p*** |
| --- | --- | --- | --- | --- | --- |
| **DUSI-R APD**  Mean(SD)  *N = 26* | 19.0(11.5) | N = 12  23.1(12.6) | N = 14  15.4(9.5) | *t*(24) =1.78 | .044* |
| **DD, AUClogd**  Mean(SD) | N = 25  .54(.19) | N =11  .56(.21) | N = 14  .52(.18) | *t*(23) = .44 | .66 |
| **BAS Drive**  Mean(SD) | 10.7(2.4)  11(6-15) | 9.33  9.5(7-12) | 9.2(2.9)  8(6-15) | *t*(25) =.14 | .89 |
| **BAS Fun-seeking**  Median(Range) | 12.15(2.2)  13(5–14) | 7.1(1.4)  7(5-9) | 8.5(2.6)  7(5-14) | *U* = 60.5 | .14 |
| **BAS Reward Responsivity**  Median(Range) | 17.78(1.6)  18(5-11) | 7.1(1.4)  7(5-9) | 7.3(1.8)  7(5-11) | *t*(25) = -.40 | .69 |
| **BIS**  Median(Range) | 15 (3.11)  15.3(11-23) | 15.7(3.2)  15(11-23) | 15.1(3.1)  15(11-22) | *t*(25) =.49 | .63 |

Note. Group comparisons for the BAS fun seeking scale used a Mann-Whitney U-test because assumption of normality for parametric testing was not met. DUSI-R APD used a one-tailed independent samples t-test. All other tests were two-tailed. *=*p*< .05. DUSI-R APD = Drug Use Screening Inventory, Revised, Absolute Problem Density; DD, AUC = delay discounting, area under the curve; BAS = Behavioral Activation System; BIS = Behavioral Inhibition System.

*2.7 WOF task behavior: Wave 2 and Wave 3 SI participants*

Initiation groups did not differ for percent of high-reward/risk selections (*Z*=70, *p*=.33). To determine whether Wave 2 compared Wave 3 participants demonstrated differences in response time (RT) for high-reward/risk versus to low-reward/risk selections, a two-way repeated measures ANOVA was used to examine the effect of group (Wave 2 SU Initiator vs. Wave 3 SU Initiator) and selection type (high-reward/risk vs. low-reward/risk) on RT. A main effect of selection type was found, with both groups demonstrating significantly slower RT in making high-risk compared to low-reward/risk selections (*F*(1,25)=31.4, *p*<.000). There was neither an effect of group (*F*(1,25)=.39, *p*=.54), nor a significant group × selection type interaction (*F*(1,25)=.06, *p*=.81). See Supplementary Table 12.

**Supplementary Table 12.** WOF task behavior for Wave 2 and Wave 3 SI participants: descriptive statistics

|  | **All Initiators** | **Wave 2 Initiator**  (~18-months  follow-up)  (*n*=12) | **Wave 3 Initiators**  (~36-months  follow-up)  (*n*=15) |
| --- | --- | --- | --- |
| **High-risk/reward selections, %**  Mean(SD)  Median (range) | 15(15)  10(1-59) | 10.8(9.3)  7.96(1.1-36.7) | 19.9(17.5)  10.11(4.5-58.9) |
| **High-risk/reward selections**  **RT (ms)**  Mean(SD) | 1240(390) | 1200(410) | 1270(390) |
| **Low-risk/reward selections**  **RT (ms)**  Mean(SD) | 990(270) | 940(260) | 1030(280) |
| **All selections**  **RT (ms)**  Mean(SD) | 1110(320) | 1070(310) | 1150(320) |

**References**

1. Ernst M, Nelson EE, McClure EB, Monk CS, Munson S, Eshel N, Zarahn E, Leibenluft E, Zametkin A, Towbin K, et al. Choice selection and reward anticipation: An fMRI study. *Neuropsychologia* (2004) 42:1585–1597. doi: 10.1016/j.neuropsychologia.2004.05.011

2. Carver CS, White TL. Behavioral inhibition, behavioral activation, and affective responses to impending reward and punishment: The BIS/BAS Scales. *J Pers Soc Psychol* (1994) 67:319–333. doi: 10.1037/0022-3514.67.2.319
